# Spatial Alignment and Response Hand in Geometric and Motion Illusions

**DOI:** 10.3389/fpsyg.2017.01169

**Published:** 2017-07-14

**Authors:** Lisa Scocchia, Michela Paroli, Natale A. Stucchi, Anna Sedda

**Affiliations:** ^1^Department of Psychology, University of Milano-Bicocca Milan, Italy; ^2^Department of Psychology, School of Social Sciences, Heriot-Watt University Edinburgh, United Kingdom

**Keywords:** Poggendorff illusion, Vanishing Point illusion, hand actions, spatial alignment effect, hand dominance, attentional dominance

## Abstract

Perception of visual illusions is susceptible to manipulation of their spatial properties. Further, illusions can sometimes affect visually guided actions, especially the movement planning phase. Remarkably, visual properties of objects related to actions, such as affordances, can prime more accurate perceptual judgements. In spite of the amount of knowledge available on affordances and on the influence of illusions on actions (or lack of thereof), virtually nothing is known about the reverse: the influence of action-related parameters on the perception of visual illusions. Here, we tested a hypothesis that the response mode (that can be linked to action-relevant features) can affect perception of the Poggendorff (geometric) and of the Vanishing Point (motion) illusion. We explored the role of hand dominance (right dominant versus left non-dominant hand) and its interaction with stimulus spatial alignment (i.e., congruency between visual stimulus and the hand used for responses). Seventeen right-handed participants performed our tasks with their right and left hands, and the stimuli were presented in regular and mirror-reversed views. It turned out that the regular version of the Poggendorff display generates a stronger illusion compared to the mirror version, and that participants are less accurate and show more variability when they use their left hand in responding to the Vanishing Point. In summary, our results show that there is a marginal effect of hand precision in motion related illusions, which is absent for geometrical illusions. In the latter, attentional anisometry seems to play a greater role in generating the illusory effect. Taken together, our findings suggest that changes in the response mode (here: manual action-related parameters) do not necessarily affect illusion perception. Therefore, although intuitively speaking there should be at least unidirectional effects of perception on action, and possible interactions between the two systems, this simple study still suggests their relative independence, except for the case when the less skilled (non-dominant) hand and arguably more deliberate responses are used.

## Introduction

In everyday life, we are often exposed to stimuli which might be misleading, or result in apparently incoherent and sometimes unstable perception of visual illusions (Gregory, 1997). Our visual systems interpret these stimuli based on the available cues in the environment and standard internal processing mechanisms, instead of relying merely on objective physical properties of objects (Hoffman, 2005). Therefore, studying visual illusions can be a powerful tool to gaining insights into the properties of the visual system (Eagleman, 2001; Scocchia et al., 2014).

Visual illusions have also been used to show that perception can sometimes influence action. For instance, illusions can affect action parameters like grip aperture when objects are placed in the configuration of a visual illusion (Milner and Dyde, 2003; Gonzalez et al., 2006; but see Van Der Kamp et al., 2012). In particular, the left hand is influenced to a greater extent by visual illusions than the right hand (Gonzalez et al., 2006). In a similar vein, the non-dominant hand is more prone to the size weight illusion (i.e., smaller objects perceived as heavier) when used to grasp and lift up objects (Buckingham et al., 2012).

Importantly, this influence of perception on action is especially seen at early stages (i.e., movement planning). For instance, during actions, congruency between the hand used and the position of the stimuli results in a facilitation effect, in other words in faster actions (De Stefani et al., 2014). On the contrary, incongruence between the hand used and the spatial position of the visual stimulus (right hand – stimulus on the left) results in an interference effect, with participants taking more time to plan and consequently to perform an action (De Stefani et al., 2014). This phenomenon is known as the spatial alignment effect, and affects human perception and performance as shown by the well-known Simon Effect (Simon and Wolf, 1963; Simon and Rudell, 1967), where responses are more accurate and reaction times faster when the stimulus appears at the same relative location as the response. Other instances of the importance of spatial alignment in human performance have been provided by evidence of facilitation in the identification of haptic stimuli by blindfolded observers facing the stimuli rather than orthogonally to them (Scocchia et al., 2009). Furthermore, visual properties of objects that are useful to plan movements are processed well in advance before execution. This is the case of affordances (Tucker and Ellis, 2004). When seeing a cup of coffee with the handle oriented in a graspable position, we are faster in both – deciding to act upon as well as executing the real movement. In a similar vein, a model trying to explain the influence of visual illusions on actions (Glover, 2002) claims that when individuals are planning a movement, influence of illusions is greater than during online control of movements, paralleling the effect seen for affordances.

Interestingly enough, the ample discussion on the influence of illusions on actions (Carey, 2001) has not been followed by the reverse: do action related properties, usually taken into account when planning a movement (even in the absence of the real movement), influence perception? One prediction that follows from the interplay between action and perception is that such an effect should be visible even when no “real action” takes place, such as in a simple adjustment task requiring a keyboard or mouse response. Even in this case, visual properties that are related to affordances should affect perception (Glover, 2002; Borghi, 2004; Tucker and Ellis, 2004). Importantly, this would suggest that the action-perception modulation in the case of illusions is bi-directional.

To shed light on this hypothesis, we explored if perception of the Poggendorff (geometric illusion) and of the Vanishing Point (VP) illusion (motion illusion) is affected by two parameters that commonly influence action planning: the hand of response and stimulus spatial alignment. The first one was picked because of its well-documented connection with perception. The second one was used because of its effect on action performance (Whitney et al., 2003). Importantly, the Poggendorff illusion significantly activates areas that are related to action and not only areas related to perception: the left premotor cortex and the left inferior frontal cortex (Shen et al., 2016). The premotor cortex is known to be involved in transforming the spatial features of perceptual stimuli into sensorimotor information useful for the action system (Shen et al., 2016). On the other hand, perception of motion, real or illusory, is related to visual activity in early occipital as well as higher-order temporal areas, which contribute more to perception than action planning (Newsome and Paré, 1988; Tootell et al., 1995).

As mentioned above, we used a simple adjustment task requiring a keyboard or a mouse response as the aim of our study is to investigate the effects of response mode (and, putatively, the impact of the mechanisms that can be critical for action planning) on perception, when no target-related action is really performed. The adjustment task has been adopted as a comparison task to a grasping task commonly used in previous studies on the influence of visual illusions, or lack of thereof, on action performance (Aglioti et al., 1995; Franz et al., 2000, 2003). Despite its motor component, this task is driven by conscious perception of a perceptual criterion rather than by biomechanical constraints or physical parameters differently from a complex movement in which the target object properties, such as size or orientation, are processed in relation to the limb performing the action (Goodale et al., 2005, 2008).

Different predictions follow the described hypothesis. Firstly, if action influences perception, hand dominance (using the left or the right hand to respond) as well as hand and spatial-alignment interactions (visual stimulus – hand congruence) might lead to different magnitudes in illusion perception (Carey, 2001). This prediction follows from previous studies in which illusions influenced more the left hand in both right and left handers (Gonzalez et al., 2006; Buckingham et al., 2012). Similarly, studies on visual affordances of objects suggest that congruency leads to a greater precision when performing a movement (Sartori et al., 2011). Translating this into illusions, one could predict a more accurate performance, in other words a smaller magnitude of the illusion, for the congruent configuration. On the other hand, if action does not influence perception, hand dominance and spatial alignment should not modify perception of illusions, suggesting a uni- rather than a bi-directional modulation.

## Materials and Methods

### Participants

Seventeen naïve participants took part in this experiment (10 males, average age and standard deviation = 29.8 ± 9.7 years). All participants were recruited at the School of Social Sciences (Psychology Department) at Heriot-Watt University (Edinburgh, United Kingdom). Only right-handed participants were enrolled in this study to avoid interferences related to brain lateralization (Ionta and Blanke, 2009). The Edinburgh Handedness Inventory – short form (Veale, 2014) was used to assess handedness, with a cut off to discriminate right handed participants of 61 (Veale, 2014). All participants had normal or corrected to normal vision and were right-handed (average laterality quotient 91/100; range: 62.5–100). Finally, participants had no history of neurological or psychiatric conditions, either chronic or degenerative, and no drug or alcohol abuse or treatment.

The study followed the guidelines of the Declaration of Helsinki and was approved by the local Ethical committee (Approval number: 2015-130). Before taking part in the study, each participant signed the informed consent and agreed to participation. The protocol was approved by the School of Life Sciences Ethic Committee.

### Stimuli

#### Poggendorff Illusion

**FIGURE 1:**
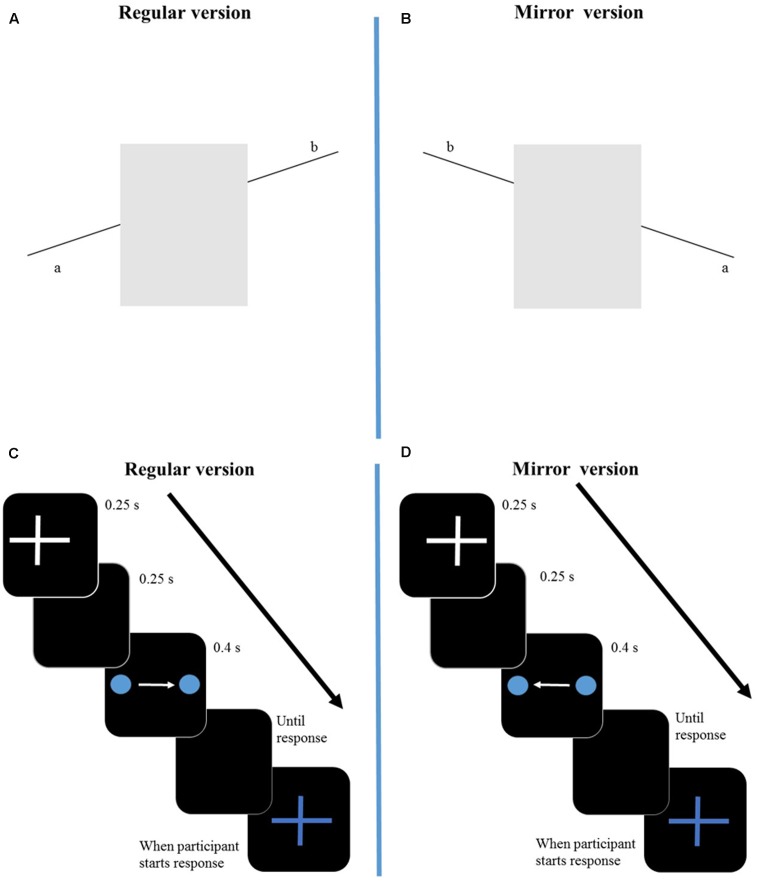
Representation of the regular version (left side) and mirror version (right side) of the two illusions. In the regular version of the Poggendorff illusion **(A)**, participants need to align the line on the right (b) with the line on the left (a). In the mirror version of the Poggendorff illusion **(B)** participants align the line on the left (b) with the line on the right (a). In the VP illusion regular version **(C)**, the dot moves from the left side to the right side, where it disappears. In the mirror version **(D)** the dot moves form the right side of the screen to the left one. Stimuli are not drawn to scale. The arrow represents time from start of trial until participant’s response. S, seconds.

The Poggendorff illusion is a geometrical illusion in which two collinear oblique line segments, which are separated by two vertical lines, are perceived as misaligned. In our stimuli (**Figure 1A**), the oblique line segments were black and measured about 1.7 cm each in length and 0.03 cm in width; they were separated by a light-gray rectangle (4.1 cm × 2.2 cm) and, therefore, in a horizontal dimension the stimulus subtended a visual angle of around 5.35 degrees. The line segments and the rectangle were presented in a 5.4 cm × 5.4 cm white inset on a gray background: the lower basis of the rectangle laid on the edge of the inset, bordering with the gray background. The stimuli were centered on the screen center.

#### Vanishing Point Illusion

The VP illusion is a visual phenomenon in which participants perceive the location where the stimulus (a dot) disappears to be displaced forward to its real position, in the same direction of the stimulus movement. In our protocol, a blue dot (diameter: 0.2 cm) (**Figure 1C**) was displayed on a black background and translated horizontally, from left to right (regular condition) or from right to left (mirror condition). Its velocity was about 9 cm/s and the length of its trajectory was 3.5 cm and, therefore, in a horizontal dimension the subtended visual angle of the displacement was around 3.99 degrees.

#### Mirror Versions of the Tasks

The mirror versions of our illusory displays were the same stimuli as described above but presented to the observer in reversed views (**Figures 1B,D**). For the Poggendorff Illusion, participants aligned the left line segment with the right line segment and for the VP participants saw a blue dot moving from the right side of the screen to the left side. The procedures and instructions were the same as the ones used with the regular versions of the stimuli.

### Procedure

The task was administered through a custom software developed with Matlab (version R2015a for Windows) and Psych Toolbox Version 3. Stimuli were presented on a 15.6 inch laptop computer with a video card ATI Mobility^TM^ Raedon^®^ HD 5145 with 512 MB DDR3 VRAM (resolution: 1366 × 768 pixels, refresh rate: 60 Hz).

Participants sat at a distance of about 50 cm from the screen, in a homogeneously ceiling illuminated and quiet room. During the experiment, participants were instructed to keep their posture still.

In both experiments, participants were required to perform an adjustment task, with both hands, one at the time. The starting hand in both tasks was randomized across participants.

In the Poggendorff Illusion experiment (**Figures 1A,B**), participants were instructed to adjust the position of one of the line segments by moving the up arrow key (to move the line up) and the down arrow key (to move the line down) to align the right line with the left line (regular version), or the left line with the right line (mirror version). A modified version of the PEST procedure (Taylor and Creelman, 1967) that has been described elsewhere (Scocchia et al., 2015) was employed for the adjustment task. The procedure can simply be described as follows: during the first adjustments of the right line, its position change was particularly evident. After these initial adjustments, this change progressively decreased in amplitude, up to a minimum distance of 1 pixel from the current and the previous position, and a green “traffic light” (a green circle, diameter: 0.7 cm) appeared on the left corner of the screen. When the participant saw the two lines segments as exactly aligned, he confirmed his response by pressing the space bar and proceeded to the next trial. Before a new stimulus was presented, a full screen mask composed of a white-noise luminance square distribution was displayed for 1 s. The task was composed of 12 trials, preceded by preliminary familiarization.

**FIGURE 2:**
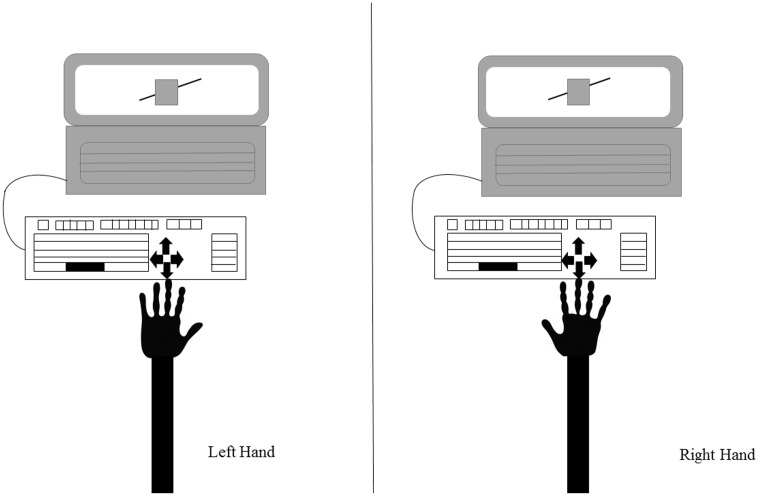
Representation of the study setup. The participant is seated in front of the laptop showing the experimental stimuli (in this example, the Poggendorff illusion). An external keyboard is attached to the laptop to ensure the body midline is not crossed. The figure shows the keyboard and the position of the arrows used to respond: as it can be seen, this setup allows responses with both hands maintaining the same spatial alignment with the stimulus.

Given that crossing the body midline, an imaginary line that divides the body into two equal parts (Holmes et al., 2006; Ceyte et al., 2007), results in an invasion of the space of the opposite side of the body that can cause spatial interference, an external keyboard was attached to the laptop (**Figure 2**). The response keys were aligned with the body midline and participants provided their responses with their index fingers, without any crossing of the body midline.

The procedure of the VP illusion experiment is illustrated in **Figures 1C,D**. At the beginning of each trial, a white crosshair composed of two lines of about 0.35 cm × 0.03 cm intersecting at their midpoint was displayed for 0.25 s on a black screen. Participants were informed that the position of the white crosshair coincided with the starting position of the vanishing dot. The presentation position of the white crosshair varied on each trial with a random (positive or negative) jitter that could range between 0 and 0.88 cm on both the *y*- and the *x*-axis. The *y*-axis jitter was centered on the screen center, whereas the *x*-axis jitter was centered 2.5 cm to the left of the center in the regular condition and 2.5 cm to the right of the center in the mirror condition. Therefore, the vanishing dot trajectory always started to the left of the center and ended to the right of it in the regular condition. Vice versa, in the mirror condition, it started to the right of the center and ended to the left of it. After the white crosshair had disappeared, a blank black screen was displayed for 0.25 s. Afterward, the vanishing dot was presented: it completed its trajectory in 0.4 s and disappeared. The blank black screen was presented until the participant moved the mouse to provide the response: at that time, a blue crosshair of the same dimensions as the starting white crosshair and of the same color as the vanishing dot was presented as mouse cursor. It was displayed only when the participant started his response movement, at a random distance in the vanishing point neighborhood (min: 1.75 cm, max: 3.5 cm, on both the *x*- and the *y*-axis, in both directions), in order to minimize external referencing to the target. Participants were instructed to place the blue crosshair exactly at the same place where they had just seen the blue dot disappear and to click the left mouse button with their index finger to record their answer. Afterward, a black blank screen was displayed for 1 s and a new trial began. The task was composed of 12 trials, preceded by preliminary familiarization.

### Data Analyses

Data have been analyzed using Matlab (Mathworks, Natick, MA, United States) and Statistica (Statsoft, Italy). A repeated measures ANOVA was conducted for both the Poggendorff illusion and for the VP illusion on two dependent measures: *illusion size* and *illusion variability. Condition* (regular, mirror) and *Hand* (left, right hand) have been introduced as within-subjects factors. Alpha level was set at 0.05. Outliers have been defined as data points below and above 2 standard deviations of the overall distribution mean, in all tasks. Partial eta squared is reported as $\eta_{p}^{2}$.

*Illusion size* indicates the magnitude of the perceived illusion. Thus, this variable is expressed as the individual constant error of adjustment with respect to the correct alignment for the Poggendorff Illusion or the real disappearing point for the VP Illusion. The average distance between the participant’s and the correct response is computed in pixels using the 12 trials composing both the experiments.

*Illusion variability* is the variable error of adjustment. This variable refers to the individual responses standard deviation (set of 12 trials), and allows to measure variability in perceiving the illusion (i.e., the greater the variability, the less homogeneous the responses).

For the VP illusion, the variables have been separately analyzed for the displacement on the *y*-axis (gravitational error) and for the displacement on the *x*-axis (horizontal displacement error).

## Results

### Poggendorff Illusion

**FIGURE 3:**
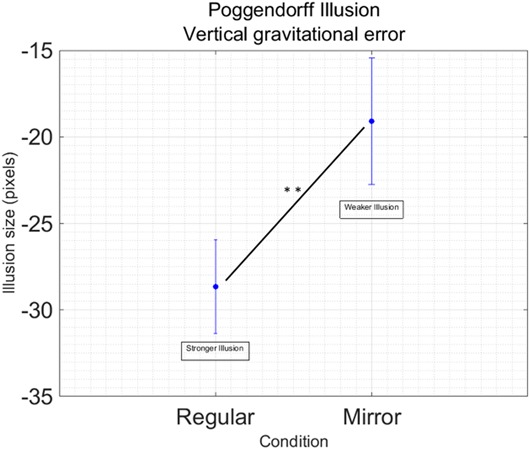
Main effect of Condition in the Poggendorff Illusion. Data are collapsed between the right and the left hand, since this factor was not significant in the main analysis Vertical bars represent standard error of the mean. Illusion size represent the dimension of the displacement in alignment. Negative values indicate the displacement is downward. Stars indicate a significant difference at *p* < 0.01.

Data processing resulted in removing 44 outliers across all the different conditions, in other words 5.39% of the total data points. We found a main effect of Condition [*F*_(1,16)_ = 10.53; *p* = 0.005; $\eta_{p}^{2}$ = 0.39] showing that the regular version of the Poggendorff illusion generates a stronger illusion compared to the mirror version (**Figure 3**). No other main effects [Hand: *F*_(1,16)_ = 0.65; *p* = 0.433] or interactions [Hand by Condition: *F*_(1,16)_ = 0.26; *p* = 0.614] have been found. No significant effects have been found for illusion variability [Hand: *F*_(1,16)_ = 0.305, *p* = 588; Condition: *F*_(1,16)_ = 1.83, *p* = 0.196; Hand by Condition: *F*_(1,16)_ = 0.587, *p* = 0.455].

### Vanishing Point

#### Gravitational Error (*Y*-Axis)

**FIGURE 4:**
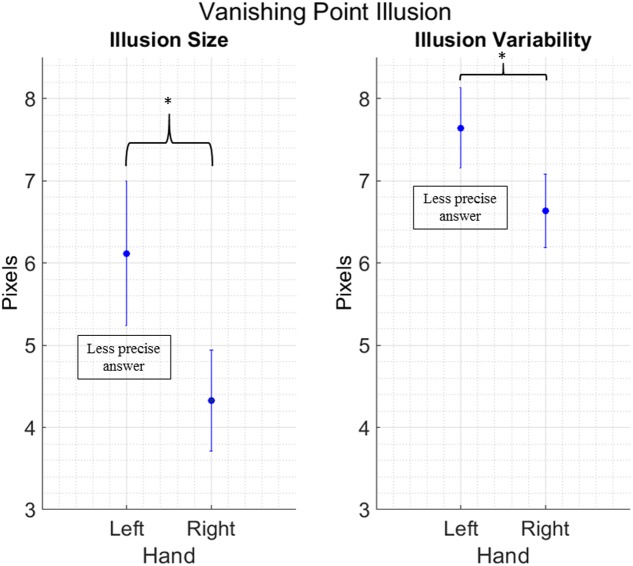
Main effect of Hand in the Vanishing Point Illusion. Data are collapsed between the regular and mirror version, as Condition did not yield any significance in the main analysis. Vertical bars represent standard error of the mean. The y axis presents error size of the vertical gravitational error. Stars indicate a significant difference at *p* < 0.05.

We removed 26 outliers across all the different conditions, in other words 3.18% of the total data points. We found a main effect of Hand for both illusion size [*F*_(1,16)_ = 7.034; *p* = 0.017; $\eta_{p}^{2}$ = 0.30] and illusion variability [*F*_(1,16)_ = 5.134; *p* = 0.038; $\eta_{p}^{2}$ = 0.24]. Both effects show that participants are less precise and more variable when they use their left hand, independently from the illusion being a regular or mirror version (**Figure 4**). No other main effects or interactions between factors have been found [Illusion size: Condition: *F*_(1,16)_ = 0.623; *p* = 0.441, Hand by Condition: *F*_(1,16)_ = 0.023; *p* = 0.882. Illusion variability: Condition: *F*_(1,16)_ = 2.550; *p* = 0.130, Hand by Condition: *F*_(1,16)_ = 0.589; *p* = 0.454].

#### Displacement Error (*X*-Axis)

Data processing resulted in removing 41 outliers across all the different conditions, 5.02% of the total data points. No significant effects have been found for this measure on illusion size or variability [Illusion size: Condition: *F*_(1,16)_ = 0.007; *p* = 0.933, Hand: *F*_(1,16)_ = 2.461; *p* = 0.136, Hand by Condition: *F*_(1,16)_ = 0.636; *p* = 0.437. Illusion variability: Condition: *F*_(1,16)_ = 0.184; *p* = 0.674, Hand: *F*_(1,16)_ = 0.003; *p* = 0.958, Hand by Condition: *F*_(1,16)_ = 0.005; *p* = 0.946].

## Discussion

Visual illusions can be generated by different cues available in the environment, such as the geometry of an image as in the Poggendorff (Koning and van Lier, 2007) or by the perception of motion as in the VP illusion (Hubbard, 1995). Both these illusions are susceptible to changes in their magnitude depending on the manipulation of their spatial properties (Actis-Grosso and Stucchi, 2003; Actis-Grosso et al., 2008; Gallace et al., 2012). Furthermore, differences have been reported in effects between the right and the left hand when actions are directed toward targets which have perceptual illusory properties (Gonzalez et al., 2006; Buckingham et al., 2012; Van Der Kamp et al., 2012).

The aim of our study was to explore possible interactions between the response mode (here: the acting hand – right vs. left) used to adjust or indicate target locations, and the arrangement of the illusory displays (regular vs. mirror reversed). Previous studies explored the effects of illusions on action, and often reported lack of thereof, but not the reverse (Goodale et al., 2008). One could hypothesize some modulatory effects of action undertaken to respond in a task on the perception of illusions themselves given the examples from the perceptual domain, such as the case of affordances, where action related properties of seen objects affect processing velocity even in absence of a real action (Glover, 2002; Borghi, 2004; Tucker and Ellis, 2004). We explored if the compatibility between the hand and the target part of the illusory stimulus has a role in reporting the extent of geometric and movement related illusions. More specifically, healthy participants were shown the Poggendorff (geometric) illusion and the VP (motion related) illusion, both in a regular and mirror version, and were asked to reply using either their right dominant or their left non-dominant hands.

Our findings show a different pattern of responses in the contexts of geometric- and motion-related illusions. The response mode did not play a role in the Poggendorff illusion. Yet, in the VP illusion, participants show more variability in reporting the illusory target location, making greater gravitational errors (displacements on the *y*-axis) when they use the left hands. This latter outcome is consistent with previous studies showing greater influence of illusory displays on the responses performed with the left (but not right) hands (Gonzalez et al., 2006; Buckingham et al., 2012), as opposed to revealing no differences between hands (Van Der Kamp et al., 2012). In our case, one could speculate that using the left hand makes a right-handed participant less accurate and increases the illusion size. However, the effect is somewhat spurious and likely related to the poorer motor control of their non-dominant (and less trained) left effector, as not only the performance accuracy, but also its precision is lower with the left hand, indicating an increase in response variability. Put it differently, the variable error, and not only the constant error, increments when participants provide their response with their non-dominant hand. As such, it seems more conservative to ascribe the results on illusion size to the participants’ tendency to shift their responses toward the bottom part of the screen (due a poorer control of the effector), rather than to a perceptual phenomenon. Secondly, for this illusion, the hand effect emerges independently form the direction of the movement being from left to right or from right to left. In other words, the spatial alignment does not affect illusion susceptibility in this motion illusion. Given that perception of motion related illusions may rely on early visual (occipital), as well as higher-order (temporal) areas, which contribute more to perception than action planning, one could conclude that action related parameters do not affect these types of illusions (Newsome and Paré, 1988; Tootell et al., 1995).

In the Poggendorff illusion, we found that the regular version generates a stronger effect, independently from any spatial alignment. Spatial alignment is seen when the hand giving the response is lateralized to the same hemifield where the stimulus is presented (i.e., left hand and left hemifield). As such, one would expect an interaction between Condition and Hand to confirm this effect. However, we only found an effect for Condition, meaning that the congruency/incongruence between the hand and the visual field does not play a role. In other words, the oblique line placed on the right side of the figure elicits a greater illusory displacement in the Poggendorff display and this is true independently from the hand used: the oblique line located on the right side of the space still generates a bigger effect even when the answer is given with the left hand. This pattern is similar to what has been previously found for the Müller-Lyer illusion, which equally impacts movement parameters of both grasping hands (Van Der Kamp et al., 2012).

Several explanations have been put forward to account for the Poggendorff illusion. For instance, the role of perceived angles formed by the oblique line encountering the parallel line (Greene, 1987) and the perceptual distortion of space between the parallel lines have been discussed (Greist-Bousquet and Schiffman, 1981). Importantly, our experimental design allows to rule out that performance in our regular and mirror Poggendorff illusion differ due to differences in the perceived angles (as they have the same acuteness in both versions) or from a distortion of the occlusive rectangle (which is the same in both conditions). Accordingly, space geometry (in particular spatial anisometry) appears to have a greater influence on the Poggendorff illusion perception than any spatial compatibility or hand effect. Asymmetries in attention direction are assumed since the first models, developed to explain neglect, in which the Authors describe two attentional vectors with a left hemispheric vector being stronger than the right one (Kinsbourne, 1970). Similarly, Corbetta et al. (1993) propose a dominance for the right hemisphere that guides attention toward both hemispaces, while the left one only toward the right hemispace. All these models, in different ways, suggest an advantage for the right hemispace that we show for the first time also for illusions, in which the target stimulus elicits a greater magnitude of the illusion when located in the right visual field. Attentional attraction toward this side of space appears to increase the perception of the illusion through anisometric perception mechanisms. Future studies could rule out if this is related to a right–left anisometry or a directional anisometry, as both explanations are possible. In other words, whether the oblique line to be adjusted is located on the right side of space might influence our ability to align it, causing a distortion of perceptual information that further increases the illusion size. This might not be the case if the line is on the left – where our perceptual system can follow a classic left-to-right direction of visual exploration – and as such might not be affected by anisometry (vertical axis anisometry). Another possibility is that oblique lines located in the right hemifield cause a directional bias as they “point” toward the lower part of the visual filed (assuming visual scanning starts from the left), while the line located in the left side of space in the mirror version points toward the upper part of the figure (top-down axis anysometry). In any case, it is relevant to highlight that visual setups taking into account attentional phenomena could prove helpful to understand illusions perception, as disagreement between theories might be due to the lack of proper manipulations that allow to weight the contribution of cognitive functions other than perception to these phenomena.

Independently from the explanation of the increase in illusion size observed in the regular versus mirror version of the Poggendorff illusion, our findings confirm that parameters taken into account when planning a “response action” do not affect perception of this illusion, suggesting lack of direct influences of perception on action, or vice versa.

In summary, our results do not support the idea that action parameters involved in reporting (or adjusting) illusory distortions, that might be also relevant for the planning of motor responses in general, can influence the magnitude of the perceived visual illusions. This result challenges the idea that similar visual representations are shared even in an initial stage of perception *per se* and planning of relevant actions (Glover, 2002). While the idea of an influence (at least to some extent) of perception on action planning holds (Carey, 2001), the reverse cannot be confirmed, at least by our experimental manipulations. Bruno (2001) exploring the discrepant results on visual illusions and action, asks “Even if dissociable at some stage, visual perception and visually planned action must coordinate at some other stage. Which one and where?” Our findings suggest that this coordination does not happen at an early stage, where independence can be seen between the two processes.

Although only right-handed participants were tested in this study to avoid any interference from possible changes in the lateralization of functions in the brain (Ionta and Blanke, 2009), the reports by Gonzalez et al. (2006) and Króliczak et al. (2016) suggest that the outcomes should not be that different for left-handers. After all, the majority of them (around 70%) should have higher-order praxis skills lateralized similarly to right-handers (Króliczak et al., 2011; see also Corballis, 2017). It is tempting to say, though, that the results obtained from the remaining 30% of left-handers could be substantially different. Such participants should be of interest for any laboratory that could selectively target such a sample of individuals in their research.

## Author Contributions

Conceived and designed the experiments: LS and AS. Performed the experiments: MP. Analyzed the data: LS. Contributed reagents/materials/analysis tools: NS and LS. Wrote the paper: LS, MP, NS, and AS.

## Conflict of Interest Statement

The authors declare that the research was conducted in the absence of any commercial or financial relationships that could be construed as a potential conflict of interest.
